# Radiography of the Appendix

**Published:** 1918-05

**Authors:** 


**Radiography of the Appendix.** M. Cohn, quoted in Arch. d'Electhicite Med., Oct., 1917, claims that the appendix is usually visible by radiography. It fills with bismuth sometime after the entrance of the latter from the ilium into the caecum, probably on account of retrograde peristalsis of the colon. It may remain filled after the caecum has cleared itself, or it may fill and empty itself several times while the caecum is filling. On account of its great motility, the appendix assumes various forms which should not be mistaken for pathologic conditions.

